# Modeling the optical properties of twisted bilayer photonic crystals

**DOI:** 10.1038/s41377-021-00601-x

**Published:** 2021-07-29

**Authors:** Haoning Tang, Fan Du, Stephen Carr, Clayton DeVault, Olivia Mello, Eric Mazur

**Affiliations:** 1 School of Engineering and Applied Sciences, Harvard University, Cambridge, MA 02138 USA; 2 Brown Theoretical Physics Center and Department of Physics, Brown University, Providence, RI 02912 USA

**Keywords:** Optical physics, Photonic devices

## Abstract

We demonstrate a photonic analog of twisted bilayer graphene that has ultra-flat photonic bands and exhibits extreme slow-light behavior. Our twisted bilayer photonic device, which has an operating wavelength in the C-band of the telecom window, uses two crystalline silicon photonic crystal slabs separated by a methyl methacrylate tunneling layer. We numerically determine the magic angle using a finite-element method and the corresponding photonic band structure, which exhibits a flat band over the entire Brillouin zone. This flat band causes the group velocity to approach zero and introduces light localization, which enhances the electromagnetic field at the expense of bandwidth. Using our original plane-wave continuum model, we find that the photonic system has a larger band asymmetry. The band structure can easily be engineered by adjusting the device geometry, giving significant freedom in the design of devices. Our work provides a fundamental understanding of the photonic properties of twisted bilayer photonic crystals and opens the door to the nanoscale-based enhancement of nonlinear effects.

## Abstract (web summary)

The twisted bilayer photonic crystals have near-zero group velocity over the Brillouin zone and provide access to light localization that is far out of the reach of conventional photonic crystals.

## Graphical abstract

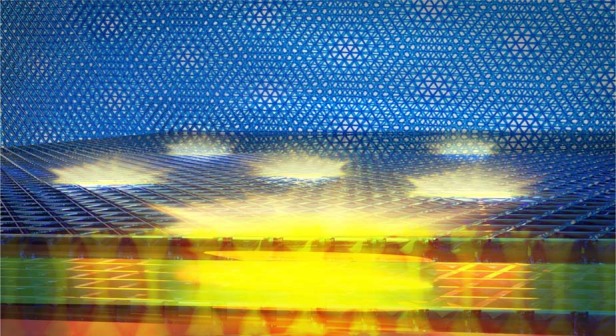

## Introduction

Over the past decade, the stacking and twisting of two-dimensional (2D) materials have led to the development of novel materials with remarkable electronic properties. For example, in twisted bilayer graphene (TBG), an engineered material consisting of two stacked layers of graphene that are rotated relative to each other, at the so-called magic angle of $\theta = 1.1^\circ$, the Fermi velocity drops to zero and the energy bands near the Fermi energy become flat^1^. These flat bands have high effective mass and half-filled correlated insulating states, resulting in superconductivity due to the formation of moiré superlattices and Dirac cone hybridization^2,3^. Exploring these unusual phenomena is central in the developing field of quantum twistronics^4,5^. The concept of twistronics has been extended to include the study of nano-light properties in materials like TBG and twisted *α*-MoO_3_^6–9^. Recently, it was shown that applying the ideas of twistronics to both 1D and 2D photonic moiré lattices in dielectric nanophotonic materials leads to slow-light effect^10,11^, light localization/delocalization phenomena^12^, and tunable resonant chiral behavior^13^. However, the connection between atomic twistronics and its nanoscale photonic analog has not been thoroughly explored.

Many concepts in condensed matter theory have photonic analogs. For example, photonic systems with nontrivial topological invariants are the photonic analog of the anomalous quantum Hall effect and the anomalous quantum spin hall effect^14–22^. The periodic dielectric lattice of honeycomb lattice photonic crystals with “artificial atoms” (the unit cell in the dielectric structure) is analogous to the hexagonal atomic lattice of graphene. Indeed, these materials have been shown to give rise to topological photonics^23–29^. In this context, it is natural to expect two layers of twisted honeycomb photonic crystal slabs to have similar physics as TBG. Yet, while the microscale analog to TBG has recently been demonstrated through phononic crystals and microwave photonic crystals^30,31^, and while tunable light properties have been observed in metamaterials with moiré patterns^32–36^, a nanoscale photonic band structure similar to the band structure in TBG-like systems has not been reported. Here, we correct that deficit.

In this paper, we report on the modeling of twisted bilayer photonic crystals (TBPhCs) consisting entirely of dielectric materials. We find that TBPhCs have a photonic band structure that is similar to the electronic band structure of TBG. At a twist angle of 1.89°, the resulting moiré flat bands have group velocities ($v_{{{\mathrm{g}}}}$) that vanish at the K point leading to an extreme slow-light effect. In analogy to the confinement of electronic wavefunctions in magic-angle TBG, we observe low-loss light localization in this linear periodic photonic system. Unlike Anderson localization in optical quasicrystals, the localization we observe does not require disorder^12,37^. As many photonic crystal and crystal analogy, TBPhCs and TBGs are that photonic states are not as tightly bound as their electronic counterparts and that the photonic system has a larger band asymmetry. The tunneling layer between the PhC slabs and the geometry of the slabs provide additional degrees of freedom for engineering the photonic band structure.

A major advantage of TBPhCs over conventional slow-light media is that TBPhCs display slow-light behavior over an extremely narrow bandwidth. We can therefore design versatile TBPhCs that operate across a broad range of visible and infrared frequencies, which can be used to realize slow-light and flat-band applications. These TBPhCs open the door to studying strong light-matter interactions, such as nanoscale-based enhancement of nonlinear effects, where a combination of light localization, low loss, and slow light is required^38–41^. In addition, they can be used to investigate flat-band phenomena and wave-packet localization in 2D systems at the nanoscale. Finally, the flexibility in designing TBPhCs permits simulating and exploring the band structure behavior of their electronic counterparts.

## Results

**Fig. 1:**
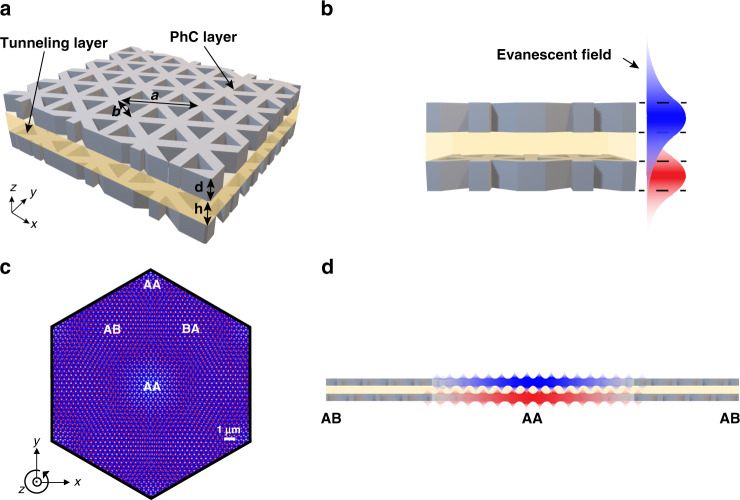
Twisted bilayer photonic crystals. **a** Bilayer photonic crystal (BPhC) consisting of a tunneling layer with a low refractive index sandwiched between two twisted dielectric layers. The 2D photonic crystal is a *d* = 220-nm-thick crystalline silicon membrane ($n_{{{{\mathrm{Si}}}}} = 3.48$) with $C_{6v}$ symmetry-protected triangular shape air holes. The triangular holes have a side length of $b = 279{\kern 1pt} {{{\mathrm{nm}}}}$ and the unit cell pitch is $a = 478{\kern 1pt} {{{\mathrm{nm}}}}$. The interlayer tunneling membrane has a thickness of $h = 250{\kern 1pt} {\mathrm{nm}}$ and the refractive index of polymethyl methacrylate (PMMA) is $n_{{{{\mathrm{PMMA}}}}} = 1.48$. **b** Two layers are separated by PMMA with only a subwavelength distance $h$, suggesting an evanescent coupling between two layers of photonic crystals. **c** Moiré pattern for a TBPhC, where the two dielectric layers are rotated by angle $\theta$ with respect to each other around the AA-stacked center. In a moiré pattern, lattice structure locally resembles the regular stacking arrangement such as AA, AB, and BA. **d** Evanescent waves from two layers of photonic crystals are mostly coupled in the AA-stacked region

**Fig. 2:**
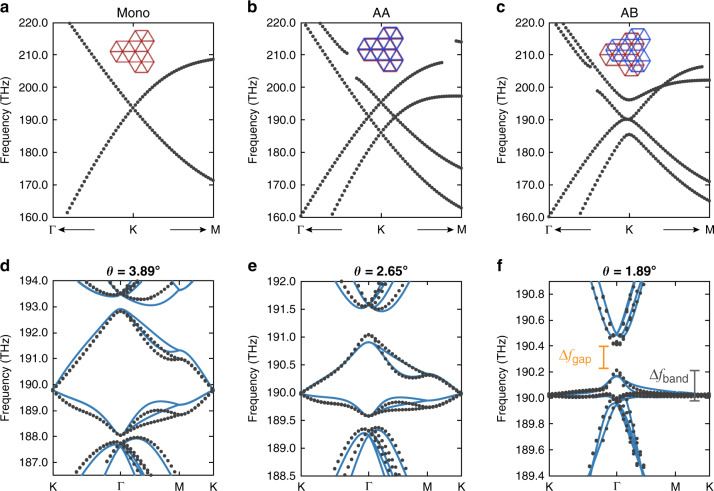
Band structures. Band structure of the monolayer (**a**), AA-stacked (**b**), and AB-stacked BPhCs (**c**). In **b**–**d**, the insets show the respective real-space configuration of the crystal unit cells. Band structure obtained from the finite-element calculation (black dots) and fitted continuum model band structure (blue line) of **d** $\theta = 3.89^\circ$, **e** $\theta = 2.65^\circ$, and **f** $\theta = 1.89^\circ$. At small angles, the Dirac cones from each layer are pushed together and hybridized due to the interlayer tunneling

Here we introduce a dielectric photonic crystal platform that hosts a band structure analogous to TBG. We start with a monolayer 2D honeycomb photonic crystal inspired by graphene^28^. The 2D photonic crystal is a silicon membrane with $C_{6v}$ symmetry-protected triangular shape air holes (Fig. 1a). By placing two photonic crystal slabs close to each other, the guided resonances in the two slabs couple through an evanescent tunneling pathway (Fig. 1b). We use a finite-element method (COMSOL Multiphysics) to numerically calculate the band structure. In the monolayer band structure, the lowest singly degenerate quasi-transverse-electric (quasi-TE) band is well isolated from other higher-order bands (see Fig. 2a). The $C_{6v}$ symmetry of the lattice also protects a Dirac-like crossing at the K point centered at the Dirac cone frequency ($f_{{{{\mathrm{DC}}}}}$), which is equivalent to the Fermi level in graphene (see Fig. 2a). In this monolayer PhC, quasi-TE electromagnetic modes that primarily propagate through air holes are weakly coupled with neighboring holes, mimicking how electrons hop between carbon atoms in graphene. The nearest- and next-nearest-neighbor coupling strength of electromagnetic modes can be controlled independently by varying the monolayer geometry, providing a platform to implement a broad class of tight-binding models. Building off this monolayer band structure, two sheets of photonic crystals are then coupled by an interlayer tunneling membrane to accurately recreate the AB- and AA-stacked configurations of bilayer graphene. In the AA-stacked configuration, two layers of PhCs are exactly aligned, while in the AB-stacked configuration, the top layer honeycomb center lies over one of the bottom layer’s triangular airhole centers. The band structure of the AA-stacked configuration looks like two copies of the monolayer bands with a vertical offset of the Dirac cones at the K point (see Fig. 2b). The AB-stacked configuration has a pair of touching parabolic bands with additional parabolic bands away from the touching bands (see Fig. 2c). Note that the AB- and BA-stacked configurations give identical band structures but not identical eigenmodes. The frequency separation between the bands in both stacking configurations is controlled by the tunneling strength between the two PhC layers, which is set by the properties of both tunneling membranes and the PhC layers.

Next, we consider two adjacent PhC layers twisted by an angle $\theta$ relative to one another. This produces a moiré pattern with a macroscopic periodicity of distinct AA and AB/BA stacking regions that grow in size as the angle decreases (Fig. 1c). Because our finite-element calculation relies on the existence of Bloch waves (see Fig. S1), we ensure that the structures created by twisting two lattices relative to each other are exactly periodic, or commensurate, by considering only specific twist angles^42^,

1 $$\theta = 2{\kern 1pt} {{\arcsin}}{\kern 1pt} {{\arcsin}}\left( {\frac{1}{{2\sqrt {3n^2 + 3n + 1} }}} \right)\quad \forall \;n \in Z^ +$$

The twist angle *θ* controls the energy scale (*E* = *hf*) at which the Dirac cones of the two PhC layers intersect in momentum space. When this energy scale is comparable to the interlayer tunneling strength, band hybridization induces moiré flat bands (see Fig. S2). The moiré flat bands are fully compressed around the Dirac cone frequency *f*_DC_ and degenerate at the superlattice K point (see Fig. 2d–f). Our TBPhCs therefore reproduce a similar band-flattening mechanism as TBG, eventually becoming flat with a zero K point group velocity ($v_{{{\mathrm{g}}}}\,({\rm K}) = 0$) at a “magic angle” of 1.89°.

**Fig. 3:**
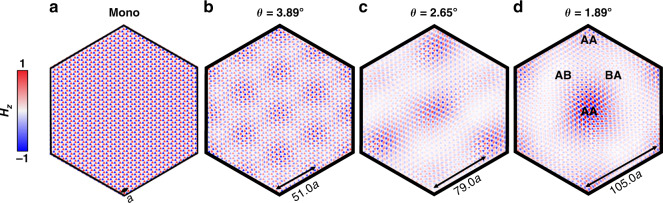
Mode localization in the twisted bilayer photonic crystals. **a** For monolayer photonic crystal slab, the quasi-TE modes at the K points are itinerant and persist across the entire supercell. **b**, **c** At large angle like 3.89° and 2.65°, the quasi-TE modes become localized on the AA-stacked region in the center of the supercell as $\theta$ decreases. **d** The quasi-TE modes are mostly localized when $\theta = 1.89^\circ$

Quasi-TE modes in the moiré bands have symmetry properties and spatial profiles that agree with electronic wavefunctions in magic-angle TBG^43^. For the monolayer PhC slab, the quasi-TE modes are located across the entire supercell (Fig. 3a). When two layers of PhC are twisted, evanescent modes are coupled more strongly in the AA site than in the AB site (Fig. 1d). For large angles, the moiré quasi-TE modes start to localize around the AA site (Fig. 3b, c). At the magic angle $\theta = 1.89^\circ$, as $v_{{{\mathrm{g}}}}\,({\rm K})$ vanishes, the quasi-TE modes become mostly localized around the AA site (Fig. 3d). This type of localization is observed over most of the Brillouin zone except at the ${{\Gamma }}$ point, where the AA site has zero-mode intensity due to the symmetry (see Fig. S3)^43^. The moiré modes, including non-flattened moiré modes, are all low-loss modes with quality factors (*Q*-factors) varying from $2 \times 10^5$ to $1 \times 10^7$. While large, these *Q*-factors are finite in contrast to the infinite *Q*-factors of the monolayer and AA/AB-stacked photonic crystals modes (see Fig. S4). The localization and high *Q*-factor properties of the moiré modes are important in the realization of device-based enhancement of nonlinear effects.

**Fig. 4:**
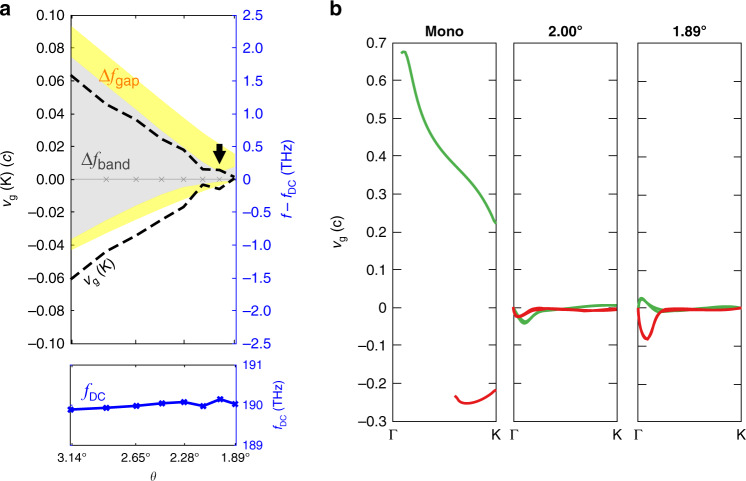
Decreased group velocity in the twisted bilayer photonic crystals. **a** Top and bottom bandgap $\Delta f_{{{{\mathrm{gap}}}}}$ (yellow), bandwidth $\Delta f_{{{{\mathrm{band}}}}}$ (gray), $v_{{{\mathrm{g}}}}\,({\rm K})$ (black dashed line), and Dirac cone frequency (blue) *f*_DC_ for small commensurate angles. The narrowest bandwidth is pointed by the black arrow. **b** Group velocity of monolayer configuration and the small-angle configuration, the lines are cut off by the light cone, the upper part of moiré bands (green) and bottom part of moiré bands (red) has different group velocity from ${{\Gamma }}$ to K

Compared to TBG’s electronic band structure, the Dirac cone frequency $f_{{{{\mathrm{DC}}}}}$ of the TBPhCs depends more strongly on twist angle and moves 0.2 THz between $3.48^\circ$ and $1.89^\circ$ (0.51% of its’ first nearest-neighbor coupling amplitude, which in TBG would correspond to a roughly 15 meV variation in Dirac cone energy). Moreover, compared to graphene, the band structure of TBPhCs has a greater asymmetry in both the moiré bandgaps (${{\Delta }}f_{{{{\mathrm{gap}}}}}$) and K point group velocities ($v_{{{\mathrm{g}}}}\,({\rm K})$) (see Fig. 2f). The bandgap above the flat band is twice as larger as that below the flat band (see Fig. 4a). We also find that at angles >$3^\circ$, the bottom bands show a much slower dispersion than the top bands.

To investigate the origin of these differences, we also calculated the band structure using a plane-wave continuum model and by considering a low-energy expansion of the TBG’s band structure. The effective Hamiltonian consists of Dirac Hamiltonians from both layers, sampled on momenta that are scattered by the moiré reciprocal lattice, and off-diagonal interlayer tunneling terms^5^. We begin with the block diagonal part, which is the Dirac Hamiltonians of each monolayer given in terms of the relative momentum *q* away from that layer’s K point ($O(q^2)$):

2 $$H_{\mathrm{gr}}\left( {{\rm K} + q} \right) \approx \frac{{ - a\left( {t_1 - 2t_3} \right)\sqrt 3 }}{2}\sigma \times q - \frac{{3a^2t_2}}{4}q^2 - 3t_2$$

where $\sigma$ is the $2 \times 2$ Pauli matrices, $a$ is the lattice constant, and the *t*_*i*_ is the *i*th nearest-neighbor couplings in a tight-binding picture for graphene. Note that although we use tight-binding coefficients to parameterize our model here, it is still a Bloch wave expansion of photonic crystal states. Some of the unconventional behavior can already be explained by this monolayer Hamiltonian. Due to relatively strong tunneling between the photonic states of the two layers, if the effective second nearest-neighbor coupling term ($t_2$) changes by a fraction of a THz as a function of twist angle, then the Dirac cone frequency *f*_DC_ will also vary with twist angle because of the last term in Eq. 2. A large $t_2$ also explains the difference between the top and bottom *ν*_g_ (K) at large angles, due to the frequency-asymmetric $q^2$ term.

We now move to the block off-diagonal terms in the effective Hamiltonian. The interlayer tunneling in TBG between pairs of orbital types of different layers (say, AA or AB) varies smoothly with the periodicity of the moiré superlattice (see Fig. 1d). This justifies their parameterization by just the first-order Fourier coefficients, commonly labeled $\omega _0$ for tunneling between orbitals of the same type (AA and BB) and $\omega _1$ for orbitals of differing types (AB and BA)^1^. To open up significant superlattice gaps, $\omega _0$ must be smaller than $\omega _1$, with $\omega _0 = 0$ maximizing the superlattice gap, while $\omega _1$ defines the effective tunneling strength for the magic-angle condition^44^. This simple model produces similar bandgaps above and below the Fermi energy. DFT calculations of lithium-intercalated TBG, however, show a large disparity in the top and bottom gaps^45^. This asymmetry was attributed to the Li atoms preferentially enhancing or screening the tunneling between the layers at different energies, affecting the effective interlayer tunneling strength for the top and bottom bands differently. As the photonic crystal states are not as tightly bound as the $p_z$ orbitals in graphene (see our estimations of the monolayer’s $t_i$ values below), having an asymmetry in the effective interlayer tunneling at high and low frequency is even less surprising here. Therefore, we fit our continuum model to the TBPhC band structures obtained by finite-element modeling by tuning variables in the following manner: for the monolayer model, we pick fixed values of $t_1$, $t_2$, and $t_3$ across all twist angles, but shift the Dirac cone frequency to a constant value; for the interlayer tunneling, we pick $\omega _0$ and $\omega _1$ independently for the top and bottom bands, giving four variables: $\omega _0^t,\;\omega _1^t,\;\omega _0^b,\;\omega _1^b$. In addition, near the magic angle, these terms should become similar, so we allow them to generically depend on $\theta$.

We find that $[t_1,t_2,t_3] = [ - 39,17, - 5]\,{\mathrm{THz}}$ works well for all twist angles. For the low-energy Hamiltonian, $v_{{{\mathrm{g}}}}\;({\rm K})$ depends on $t_1 - 2t_3$; the asymmetry in $v_{{{\mathrm{g}}}}\;({\rm K})$ sets the strength of $t_2$, so we increase $t_1$ and $t_3$ together to give a sequence of couplings that show reasonable decay in strength. In contrast, for TBG the coupling strengths given by DFT simulations decay by roughly a factor of 10 between $t_1$ and $t_2$^46^, indicating that these electronic states are much more tightly bound than their photonic counterparts.

At the magic angle ($\theta = 1.89^\circ$) we find good agreement when selecting $\omega _0^t = 1.43$ and $\omega _1^t = 1.85{\kern 1pt} {{{\mathrm{THz}}}}$ for the top-side tunneling (above Dirac frequency) and $\omega _0^t = \omega _1^b = 1.85{\kern 1pt} {{{\mathrm{THz}}}}$ for the bottom-side tunneling (below Dirac frequency). We obtain a good fit to the bands using a linear dependence of the tunneling strengths on the twist angle

3 $$\omega _i^t\left( \theta \right) = \left[ {1 - 0.15\left( {\theta - \theta _m} \right)} \right]\omega _i^t\left( {\theta _m} \right)\omega _i^b\left( \theta \right) = \left[ {1 + 0.15\left( {\theta - \theta _m} \right)} \right]\omega _i^b(\theta _m)$$

for $\theta$ evaluated in degrees. At $\theta = 4^\circ$, the tunneling coefficients are roughly 30% weaker for the top side and 30% stronger for the bottom side. The enhancement of the bottom-side tunneling is counterbalanced by a suppression of the top-side tunneling, implying that the “net” tunneling is unchanged, while its distribution between the relevant orbitals is modified, in agreement with the study of Li-intercalated TBG. This unusual interlayer tunneling behavior results in the severe top band and bottom band asymmetry we observe in the TBPhC’s band structure.

Our model gives a very reliable reproduction of the bands close to the Dirac cone frequency, with only small disagreements occurring in the top and bottom parts of the flat bands at the magic angle (see Fig. 2d–f). This is due to terms not captured by our simple model. Specifically, we omit the momentum dependence of the interlayer tunneling terms^47,48^ that provide a more accurate description of their Fourier transform.

The photonic moiré flat bands exhibit slow-light effects both at the magic angle *θ* = 189° and at *θ* = 2°. Figure 4b shows the group velocity (*ν*_g_ = d*ω*/d*k*) is drastically reduced in the **Γ** to K direction. While *ν*_g_ = 0.2*c*–0.68*c* in a monolayer PhC, in the TBPhCs at *θ* = 2°*ν*_g_ is reduced to 0.005*c* near the K point and never exceeds 0.04*c* over the entire Brioullin zone. At the magic angle *θ* = 189°, *ν*_g_ is reduced to zero at the K point and never exceeds 0.08*c*. Note that although the magic angle is at *θ* = 189°, because of the higher *ν*_g_ around **Γ** point at 1.89°, the narrowest moiré bandwidth, $\Delta f_{{{{\mathrm{band}}}}} = 0.217\,{\mathrm{THz}}$, is obtained at *θ* = 2° (see Fig. 3a). The small *ν*_g_ over the entire Brillouin zone is essential for all-directional photonic devices and any device-based enhancement of nonlinear effects.

**Fig. 5:**
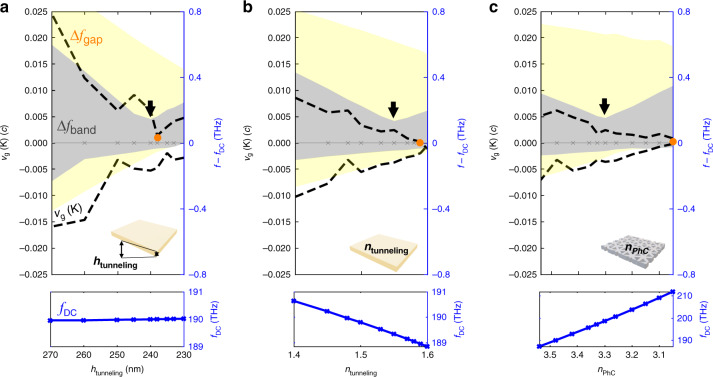
Bandstructure engineering. **a** Top and bottom bandgap $\Delta f_{{{{\mathrm{gap}}}}}$ (yellow), bandwidth $\Delta f_{{{{\mathrm{band}}}}}$ (gray), group velocity at K point $v_{{{\mathrm{g}}}}\;({{{\mathrm{K}}}})$ (black dashed line), and Dirac cone frequency *f*_DC_ when changing **a** $h$, **b** $n_{{{{\mathrm{tunneling}}}}}$, and **c** $n_{{{{\mathrm{PhC}}}}}$ . The narrowest bandwidth is pointed by the black arrow and the vanishing $v_{{{\mathrm{g}}}}\;({{{\mathrm{K}}}})$ is labeled by the orange dot

We also studied the dependence of the TBPhC band structure on the geometry parameters. We fix $\theta = 2.13^\circ$ and individually vary the tunneling layer thickness (*h*), the refractive index of the tunneling layer ($n_{{{{\mathrm{tunneling}}}}}$), and the refractive index of the PhC bilayer ($n_{{{{\mathrm{PhC}}}}}$). Varying *h*, we find that $f_{{{{\mathrm{DC}}}}}$ remains unchanged and obtain a minimum in $v_{{{\mathrm{g}}}}\;({\rm K})$ at $h = 250{\kern 1pt} {{{\mathrm{nm}}}}$ and the narrowest bandwidth $\Delta f_{{{{\mathrm{band}}}}} = 0.185{\kern 1pt} {{{\mathrm{THz}}}}$ at $h = 240{\kern 1pt} {{{\mathrm{nm}}}}$ (see Fig. 5a). Increasing $n_{{{{\mathrm{tunneling}}}}}$ decreases $f_{{{{\mathrm{DC}}}}}$, and $v_{{{\mathrm{g}}}}\;({\rm K})$ is reduced to zero when $n_{{{{\mathrm{tunneling}}}}} = 1.59$, while the narrowest bandwidth $\Delta f_{{{{\mathrm{band}}}}} = 0.18{\kern 1pt} {{{\mathrm{THz}}}}$ is obtained at $n_{{{{\mathrm{tunneling}}}}} = 1.55$ (see Fig. 5b). Decreasing $n_{{{{\mathrm{PhC}}}}}$ increases $f_{{{{\mathrm{DC}}}}}$, and $v_{{{\mathrm{g}}}}\;({\rm K})$ is reduced to zero when $n_{\mathrm{Si}} = 3.05$, while the narrowest bandwidth $\Delta f_{{{{\mathrm{band}}}}} = 0.2{\kern 1pt} {{{\mathrm{THz}}}}$ is obtained at $n_{{{{\mathrm{PhC}}}}} = 3.3$ (see Fig. 5c). In all three cases, the Dirac cone frequency $f_{{{{\mathrm{DC}}}}}$ is modified due to the change in the effective refractive index of the TBPhC. To study how the interlayer tunneling depends on the three geometry parameters, we again fitted the continuum model to the band structures obtained from finite-element modeling (wee Fig. S5). Starting from the original values of $h = 250{\kern 1pt} {{{\mathrm{nm}}}},\;n_{{{{\mathrm{tunneling}}}}} = 1.48$, and $n_{{{{\mathrm{PhC}}}}} = 3.48$, and we obtain the following dependence of the tunneling parameters $\omega _0^t,\;\omega _1^t,\;\omega _0^b,\;\omega _1^b$ on the three geometry parameters:

4 $$\begin{array}{l}\omega _1^b = \omega _1^b(\theta _m)\\ \omega _i^j = \beta \omega _i^j(\theta _m)\end{array}$$

with

5 $$\begin{array}{l}\qquad\quad\beta \left( h \right) = 1 - \frac{{h - 250{\kern 1pt} {{{\mathrm{nm}}}}}}{{135{\kern 1pt} {{{\mathrm{nm}}}}}}\\ \,\,\,\quad\beta \left( {n_{{{{\mathrm{PhC}}}}}} \right) = 1 - 0.85(n_{{{{\mathrm{PhC}}}}} - 3.48)\\ \beta \left( {n_{{{{\mathrm{tunneling}}}}}} \right) = 1 + (n_{{{{\mathrm{tunneling}}}}} - 1.5)\end{array}$$

Changing the geometry parameters affects all tunneling parameters except the AA orbital tunneling represented by $\omega _1^b$. As *h* or $n_{{{{\mathrm{tunneling}}}}}$ increases or $n_{{{{\mathrm{PhC}}}}}$ decreases, the strength of the tunneling becomes smaller. It is possible that the change in these parameters modifies not only the $\omega$ terms but also the in-plane couplings *t*_*i*_, but near the magic angle, the band structure is predominantly defined by a ratio between these two types of model parameters^1^; therefore, we consider the *t*_*i*_ fixed for simplicity. The parameter independence of $\omega _1^b$ is motivated by observations of the near parameter independence of the bottom bands, which is likely because the geometry parameters predominantly modify the higher frequency photonic modes (see Fig. S6). Understanding the dependence of the band structure on the geometry parameters provides additional degrees of freedom in further engineering the optical moiré flat band and indicates the possibility of having a magic angle at higher $\theta$, where localized modes are closer to each other in distance^30^.

## Discussion

Our numerical calculations show that the dispersion of electromagnetic waves can be manipulated dramatically, from highly dispersive to flat, by simply changing the angle between two photonic crystal slabs. We identified the magic angle where moiré flat bands appear, leading to a large reduction in group velocity compared to monolayer PhC. At this angle, TBPhCs exhibit slow-light behavior within an extremely narrow bandwidth and the eigenmodes are highly localized in the regions exhibiting AA stacking. We studied the photonic band structure behavior using a plane-wave continuum model and found that TBPhCs differ from TBG both in intralayer coupling and interlayer tunneling characteristics. We find that interlayer tunneling can be controlled by tuning the geometry parameters $(h,n_{{{{\mathrm{tunneling}}}}},n_{{{{\mathrm{PhC}}}}})$, facilitating the design of an optical flat band.

The “twisted photonic crystal toolkit” we present here provides access to slow-light effects and light localization that cannot be accomplished by conventional photonic crystals. Therefore, TBPhCs will drastically enhance access to optical nonlinearities and quantum interactions in photonic devices. Because TBPhCs are designed for standard silicon-on-substrate wafers and can be fabricated by a wafer bonding and transferring technique, the fabrication of such devices is immediately feasible.

## Materials and methods

### Simulation

The finite-element band structure, eigenmode, and *Q*-factor simulations were computed using three-dimensional finite-element methods (COMSOL Multiphysics 5.4). We first calculated all the modes in a PhC unit cell/super unit cell with Floquet periodic boundary conditions in the two lattice–vector directions and perfectly matched layers at the boundaries in the out-of-plane direction. TM/TE-polarized modes were selected by evaluating the energy ratio of the electric and magnetic fields in all directions. The simulations were carried out on a Dell M630 computer (2 × Intel Xeon CPU E5-2697 v4 2.30 GHz 18 core, 247 Gb RAM, 1 GbE, FDR Infiniband). The time to calculate the photonic band structure at the magic angle is roughly 24 h. The plane-wave continuum model is implemented using MATLAB. The estimated calculation time is a few seconds per band structure.

## Supplementary information

Supplementary Information for Modeling the Optical Properties of Twisted Bilayer Photonic Crystals

Band Compressing
