# Supplementary material for: Modeling the optical properties of twisted bilayer photonic crystals
Source: Light Sci Appl. 2021 Jul 29;10:157. doi: 10.1038/s41377-021-00601-x (PMC8322106; doi:10.1038/s41377-021-00601-x)
Supplement: Supplementary file 1 — Supplementary Information for Modeling the Optical Properties of Twisted Bilayer Photonic Crystals [file 41377_2021_601_MOESM1_ESM.docx]

**Supplementary Information for Modeling the Optical Properties of Twisted Bilayer Photonic Crystals**

**Haoning Tang^1^, Fan Du^1^, Stephen Carr^2^, Clayton DeVault^1^, Olivia Mello^1^, and Eric Mazur^1*^**

^1^ School of Engineering and Applied Sciences, Harvard University, Cambridge, MA 02138, USA

^2^ Brown Theoretical Physics Center and Department of Physics, Brown University, Providence, Rhode Island 02912, USA

^*^ Email: [mazur@seas.harvard.edu](mailto:mazur@seas.harvard.edu)

**Section 1. Simplified continuum model**

A simplified continuum model of bilayer graphene can be understood by beginning with a nearest-neighbor tight-binding model. This model consists of two Carbon atoms of the honeycomb lattice of graphene, with lattice vectors $a_{1}=a\left( \frac{\sqrt{3}}{2},-\frac{1}{2} \right), a_{2}=a\left( \frac{\sqrt{3}}{2},\frac{1}{2} \right)$ and reciprocal vectors $b_{1}=\frac{2\pi}{a}\left( \frac{1}{\sqrt{3}},-1 \right), b_{2}=\frac{2\pi}{a}\left( \frac{1}{\sqrt{3}},1 \right)$. The $A$ and $B$ sublattice atoms are positioned at 0 and $\frac{1}{3}(a_{1}+a_{2})$, respectively. Assuming a nearest neighbor coupling $t_{1}$, a second-nearest neighbor coupling of $t_{2}$, and a third-nearest neighbor coupling of $t_{3}$, the low-energy Hamiltonian for a momentum $K+q$ near the Dirac cone $K=\frac{b_{1}-b_{2}}{3}=\frac{2\pi}{a}(0,-2/3)$ is, up to ($O(q^{2})$):

$H_{gr}\left( K+q \right)\approx\frac{-a\left( t_{1}-2t_{3} \right)\sqrt{3}}{2}\sigma\cdot q-\frac{3a^{2}t_{2}}{4}q^{2}-3t_{2}$ (1)

where $\sigma$ are the $2\times2$ Pauli matrices, taken as a rank 3 tensor acting on the momentum vector $q$. The group velocity at $K$ (slope of the bands at the cone) is $v_{g}(K)=\frac{a\left( t_{1}-2t_{3} \right)\sqrt{3}}{2}$. There is also a top-bottom band symmetry-breaking term of strength $\frac{3a^{2}t_{2}}{4}$, which goes like $q^{2}$ along the diagonal of $H$, causing both top and bottom levels away from $K$ point to be pushed in the same direction.

The interlayer coupling between two graphene monolayers can also be highly simplified. The coupling between any pair of interlayer orbital types can be represented with a first-order Fourier expansion, consisting of three plane-waves of equal strength summed together, and with the relative phases of each term chosen appropriately such that the maxima are centered at the appropriate location in the moiré superlattice. This introduces $2\times2$ scattering matrices with specific phases for the three-fold related scattering momenta, namely, ^1^

$T_{1}=\left( \begin{aligned} \omega_{0} \omega_{1} \\ \omega_{1} \omega_{0} \end{aligned} \right), T_{2}=\left( \begin{aligned} \omega_{0}\psi^{*} \omega_{1} \\ \omega_{1}\psi\omega_{0}\psi^{*} \end{aligned} \right), T_{3}={T_{2}}^{*}$ (2)

for $\psi=e^{i2\pi/3}$.

The bilayer Hamiltonian can then be easily formulated by combining these two simplifications:

$H(k)=\left( \begin{aligned} H_{D}\left( q \right) \Sigma T_{i} \\ \Sigma T_{i}^{\dagger} H_{D}(q) \end{aligned} \right)$ (3)

where $k$ is taken nearby the $K$ point of monolayer graphene’s Brillouin zone. The $T_{i}$ scatter from the given $k$ to nearby, but different $q$, determined by the reciprocal vectors of the moiré superlattice ($q_{1} = 0$, $q_{2} = G_{2}$, and $q_{3} =-G_{1}$, for $G_{i}$ the reciprocal vectors of the moiré supercell given in Fig S. 1).

**Section 2.** **Inter-layer versus intra-layer modifications**

In twisted-bilayer graphene, the “magic-angle” phenomena is (roughly) described by a matching of inter-layer and intra-layer coupling strengths. The normalized velocity of bands equals to,

$\frac{v^{*}}{v}=\frac{1-3\alpha^{2}}{1+6\alpha^{2}}$ (4)

Where $\alpha=w/vk_{0}$, $w$ is the hopping energy and $v$ is the frequency^1^. When the in-plane dispersion (Dirac cone) is exactly compensated by the inter-layer hybridization (due to the moiré interface), the bands undergo an inversion during which very low dispersion is achieved. From this perspective, the continuum model cannot easily distinguish between increasing the interlayer coupling strength (making the hybridization stronger) and decreasing in the intralayer coupling strength (making the Dirac cone less dispersive), as *both* will move the magic-angle condition to a larger twist angle. On-top of this, the low-energy spectra of both situations are indistinguishable, with only the dispersion of the bands at higher energies giving hints as to which of the two situations has occurred.

Since the COMSOL simulations give well-confined H_z_ bands only near the flat-band manifold, distinguishing between the two is effectively impossible in this work, and so we have taken the approach that most closely matches the case in the electronic system to make comparisons to TBG as easy as possible. Even when making this approximation, we still see significant differences compared to electronic TBG: the effective tunneling strength changes drastically with the twist-angle, and is not particle-hole symmetric (e.g. the tunneling above and below the Dirac cone seem to change independently). We note that if instead we took the route of modifying the *in-plane* couplings as a function of twist-angle, a good reproduction of the COMSOL results could still be achieved. A mixture of the two extremes, having both the intra-layer and inter-layer terms vary with the twist angle, is also completely possible.


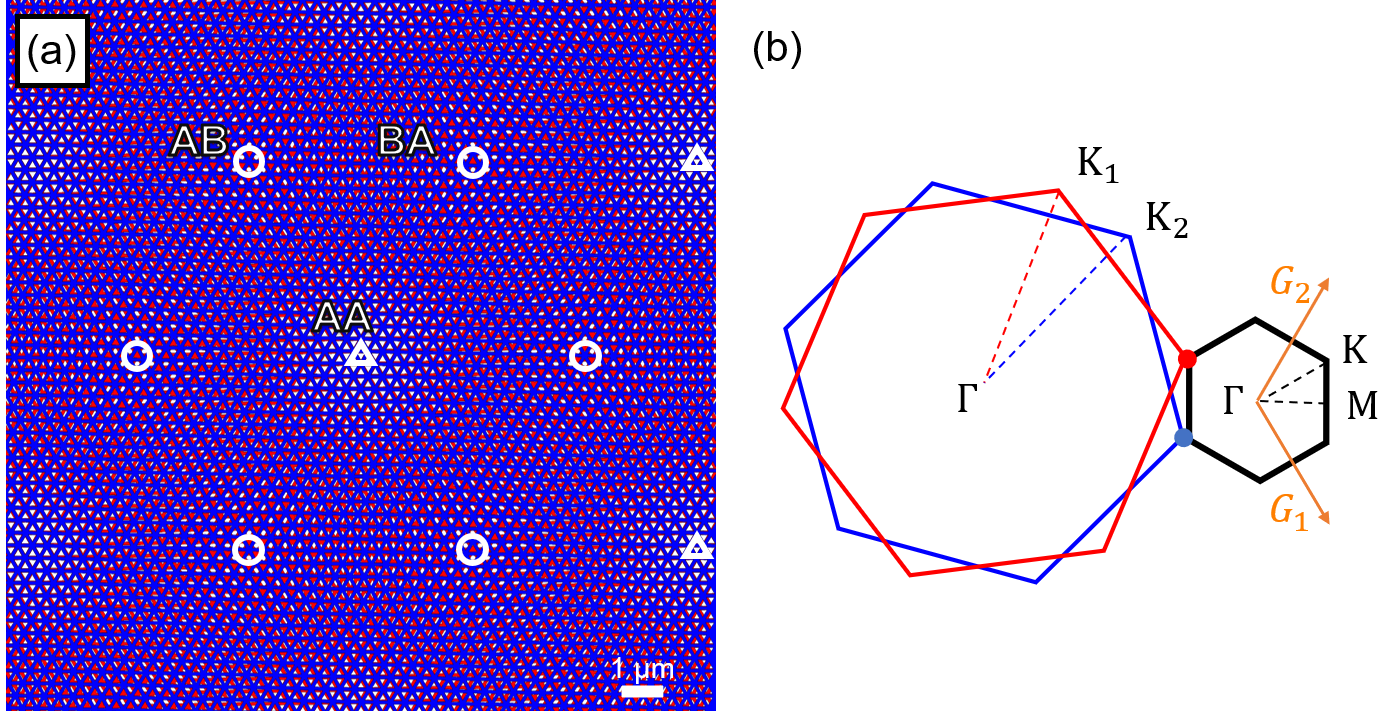


Figure S1 (a) Photonic crystal structure and moiré pattern of TBPhCs with $\theta=1.89^{\circ}$ , the top(bottom) layer is blue(red). AA and AB (or BA), are indicated by triangles and circles, respectively. The triangular superlattice vectors $a_{1}$and $a_{2}$are triangular superlattice vectors. (b)The super lattice Brillouin zone is constructed from the wave vectors of the two photonic crystals layers. and the small hexagon is the moiré Brillouin zone of TBPhCs with reciprocal vectors $b_{1}$and $b_{2}$.


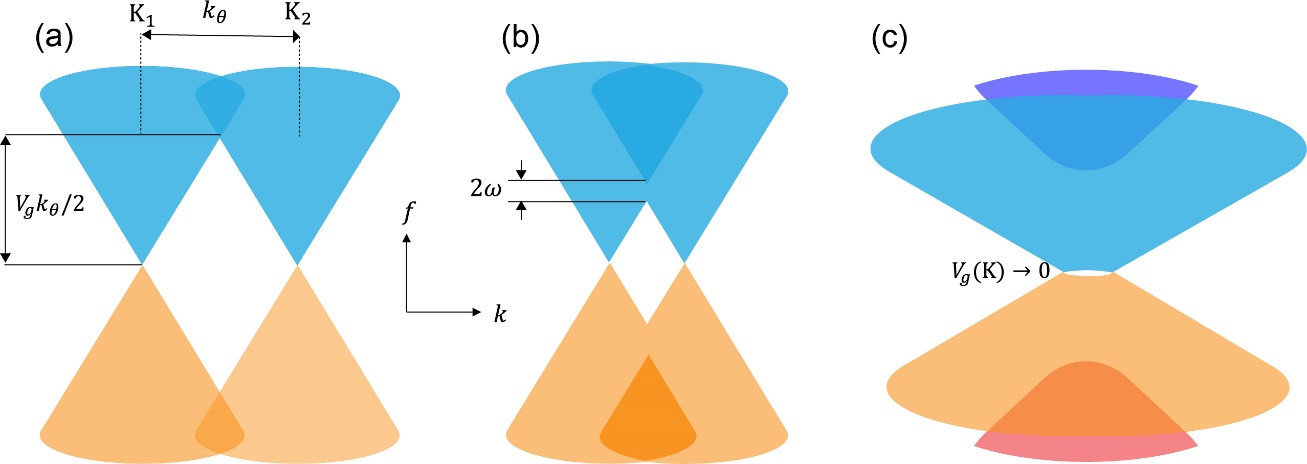


Figure S2 Rotation of the Brillouin zones causes the Dirac cones of the two layers to rotate and intersect with each other. (a) The hybridization strength is $\omega=0$ (b) When there is enough electronic tunneling between the two layers, the Dirac-cone bands hybridize with each other. Here, $2\omega\ll v_{g}k_{\theta}/2$ (c) Finally, the Dirac cone bands get closer to each other, and their hybridization becomes flat at $2\omega\sim v_{g}k_{\theta}/2$. The flattening of the bands near the magic angle can be understood as a compensation between the kinetic energy and the interlayer hybridization energy.


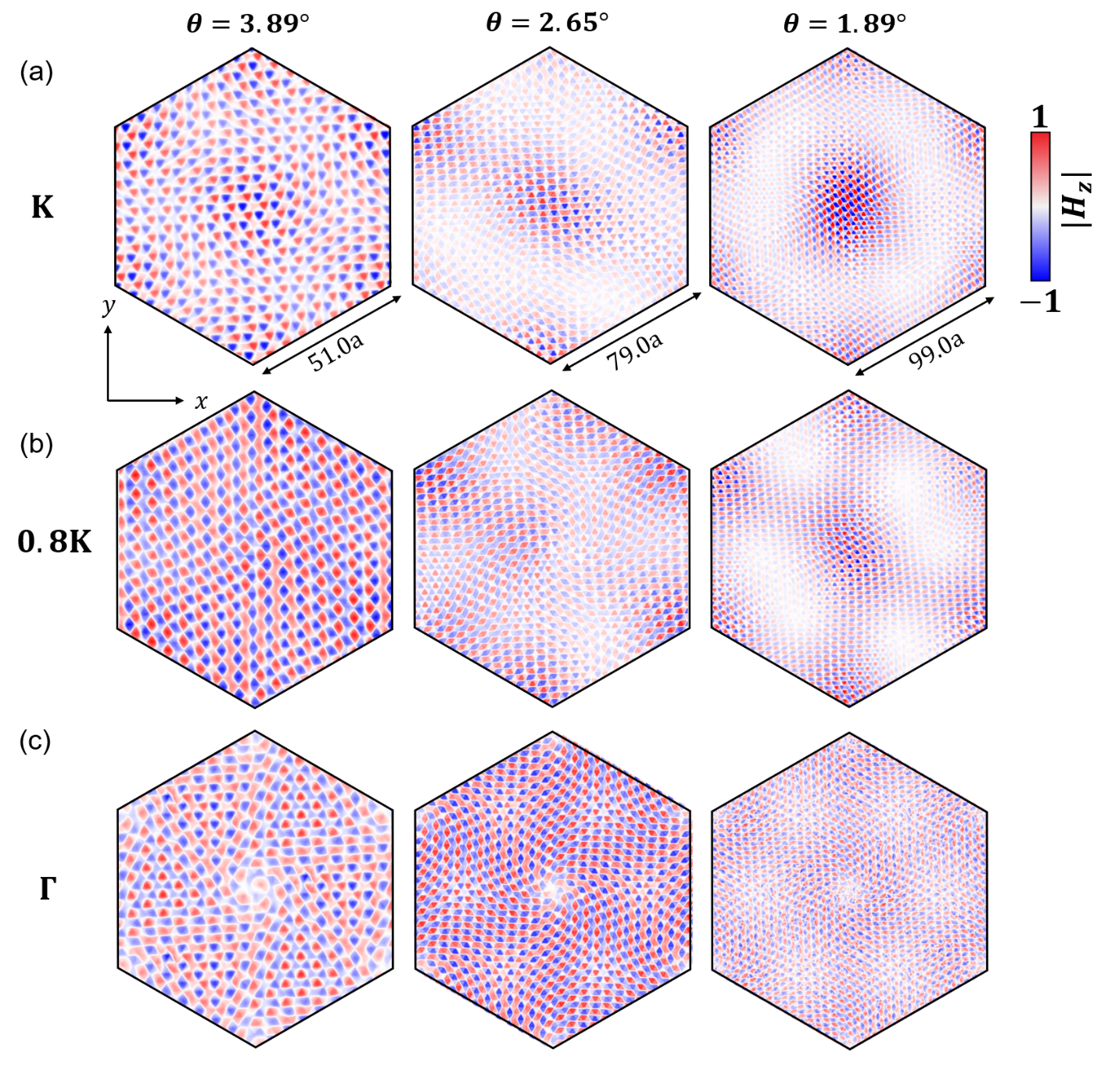


Figure S3 Eigenmode localization at (a)$K$ (b)$0.8 K$ and (c) $\Gamma$ point. At small angles, the modes are localized around the AA stacked region. Due to symmetry, the AA site has zero weight from the $\Gamma$ wavefunction.


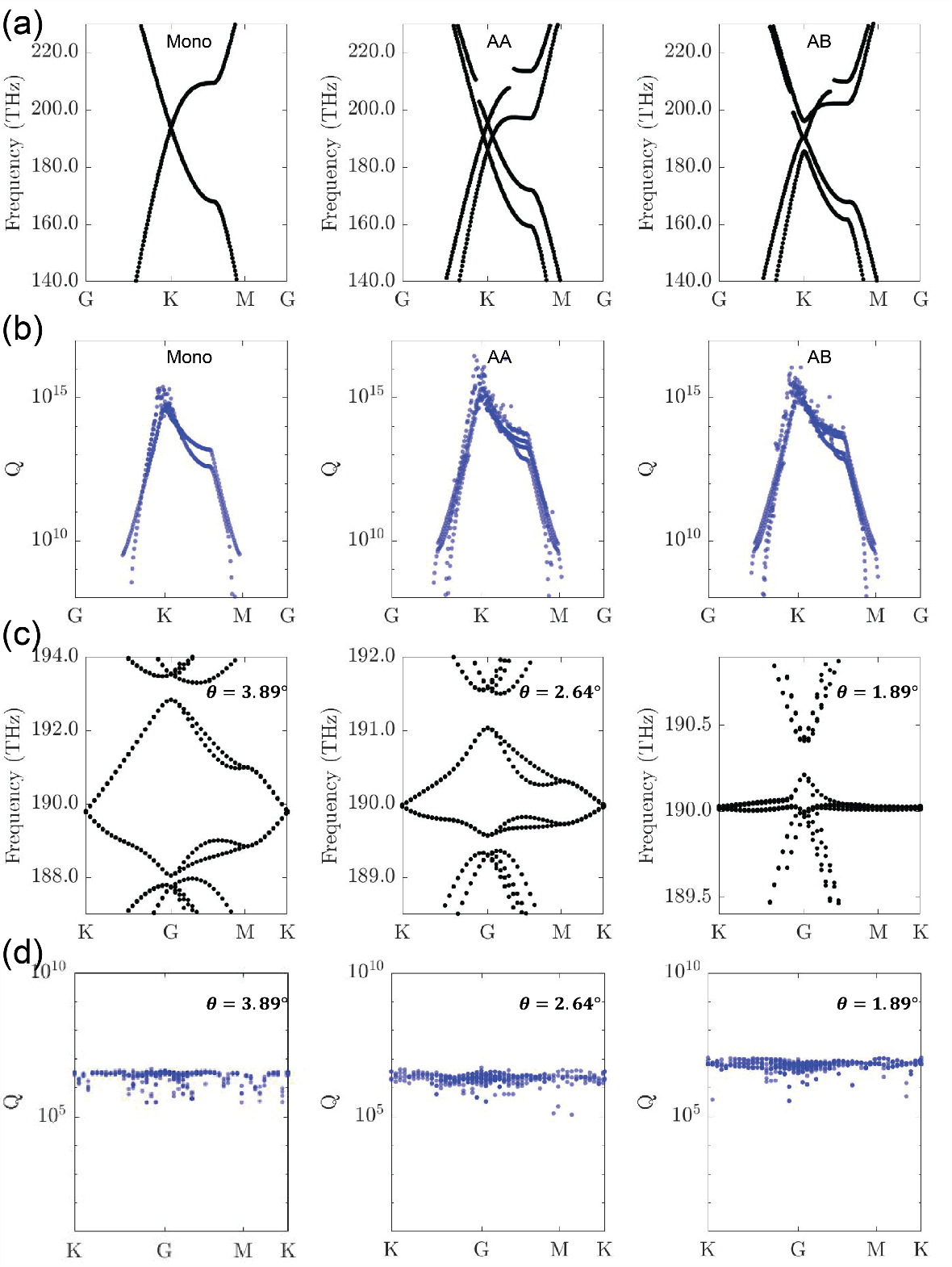


Figure S4 (a) The bandstructure and (b) quality factors ($Q$-factor) of the monolayer PhC, AA-stacked PhCs and AB-stacked PhCs are very high over the entire Brillouin zone and is infinite near the $K$ point. (c) The bandstructure and (d) quality factors ($Q$-factor) of the TBPhCs are high (${2\times10}^{5}$ to ${3\times10}^{7}$) over the entire Brillouin zone but not infinite.


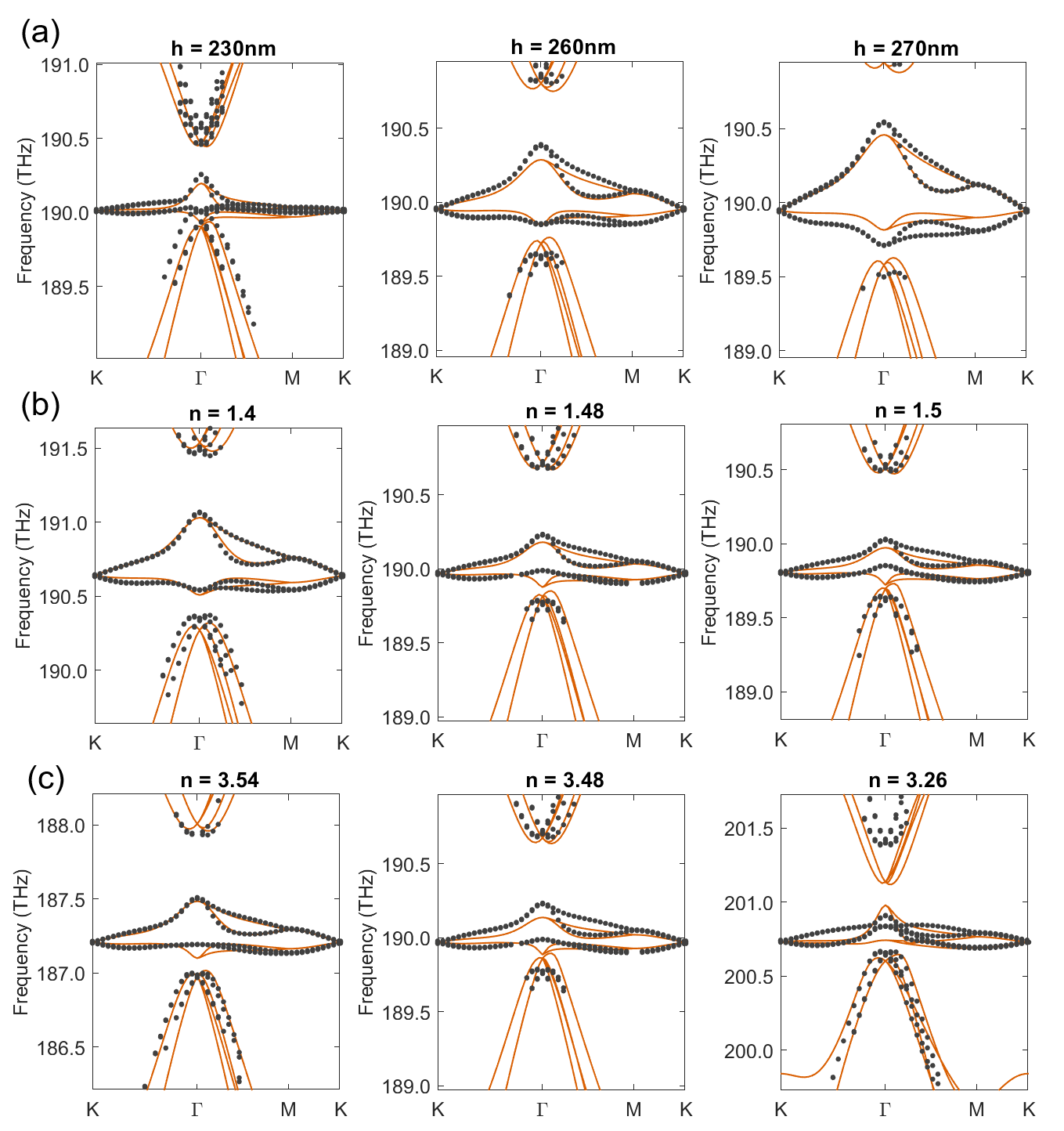


Figure S5 (a) Fitting of the continuum model (lines) to the FEM results (black dots) for changes in (a) $h$ (b)$n_{\mathrm{tunneling}}$, and (c) $n_{\mathrm{PhC}}$


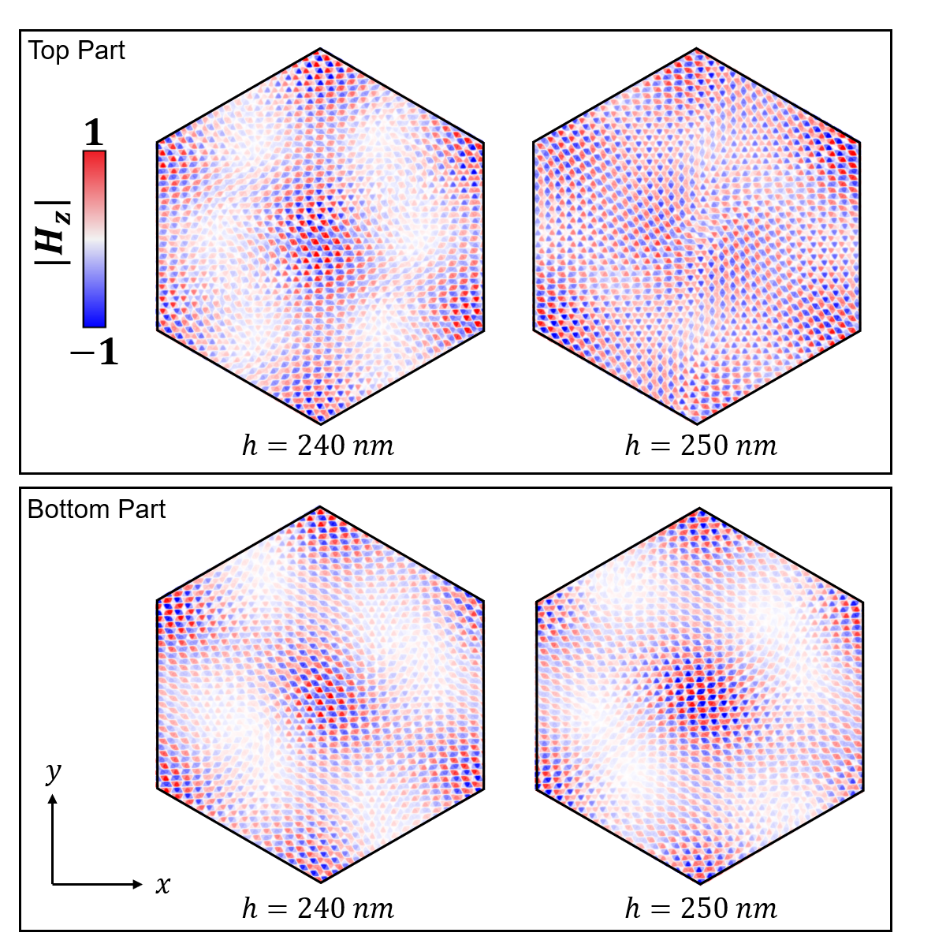


Figure S6 Geometry parameters like the thickness of the tunneling layer mostly modify the higher frequency photonic modes in the top part of moiré bands. Changing the geometry parameters does not modify the mode configuration of the bottom part of the moiré bands very much.

**Reference**

1 Bistritzer, R. & MacDonald, A. H. Moire bands in twisted double-layer graphene. *Proc Natl Acad Sci U S A* **108**, 12233-12237, doi:10.1073/pnas.1108174108 (2011).
